# Multiplex profiling of serum proteins in solution using barcoded antibody fragments and next generation sequencing

**DOI:** 10.1038/s42003-020-1068-0

**Published:** 2020-07-03

**Authors:** Mattias Brofelth, Anna Isinger Ekstrand, Shashank Gour, Ronnie Jansson, My Hedhammar, Björn Elleby, Anders Kvist, Christer Wingren, Ulrika Axelsson, Carl A. K. Borrebaeck

**Affiliations:** 1 Department of Immunotechnology, Lund University, Medicon Village, Lund, Sweden; 2 CREATE Health Translational Cancer Center, Lund University, Medicon Village, Lund, Sweden; 3 Department of Protein Science, School of Engineering Sciences in Chemistry, Biotechnology and Health, KTH Royal Institute of Technology, Stockholm, Sweden; 4 Immunovia AB, Medicon Village, Lund, Sweden; 5 Division of Oncology and Pathology, Department of Clinical Sciences Lund, Lund University Cancer Center, Lund University, Lund, Sweden

**Keywords:** Assay systems, Proteomics

## Abstract

The composition of serum proteins is reflecting the current health status and can, with the right tools, be used to detect early signs of disease, such as an emerging cancer. An earlier diagnosis of cancer would greatly increase the chance of an improved outcome for the patients. However, there is still an unmet need for proficient tools to decipher the information in the blood proteome, which calls for further technological development. Here, we present a proof-of-concept study that demonstrates an alternative approach for multiplexed protein profiling of serum samples in solution, using DNA barcoded scFv antibody fragments and next generation sequencing. The outcome shows high accuracy when discriminating samples derived from pancreatic cancer patients and healthy controls and represents a scalable alternative for serum analysis.

## Abstract (web summary)

Brofelth, Ekstrand et al use DNA barcoded scFv antibody fragments and next generation sequencing for multiplex profiling of proteins in serum from pancreatic cancer patients with high accuracy. This approach can potentially be used in high throughput precision diagnosis.

## Introduction

Human serum is a complex proteome to analyze, providing major technological challenges. However, mining the serum proteome for differentially expressed molecular biomarkers provides an attractive and minimally invasive way for precision diagnostics^1^. Planar antibody microarray is one of the technologies in the forefront^2–5^ and has delivered clinically actionable information for differential and early diagnosis of cancer^6–8^. Although highly sensitive for multiplexed protein expression profiling, planar antibody arrays strive with inherent limitations such as surface performance, signal-to-noise ratio, limit of detection, dynamic range, and printing logistics.

A solution-based platform could circumvent several limitations but has so far not been developed for the serum proteome to achieve both the necessary sensitivity and scalability. Conventional technologies are limited in target multiplexity, partly by the need of multiple antibodies per target analyte^9^. Alternative approaches have, however, been developed in recent years utilizing antibody–DNA conjugates allowing multiplexed protein analysis of fine needle aspirate using NanoString nCounter®^10^, high-throughput phenotyping of cells using next-generation sequencing (NGS)^11–14^, as well as more focused approaches using, e.g., DNA-binding factors^15^. Assays can, however, be designed using multi-well plates in automated systems for parallel and consistent serum analysis in solution, which in combination with NGS could reach ultra-high sensitivity.

Here we present a proof-of concept study for profiling serum from pancreatic cancer patients, using ProMIS, *Pro*tein detection using *M*ultiplex *I*mmunoassay in *S*olution. ProMIS is a streamlined platform for profiling of serum proteins with a solution-based bead array. The assay utilizes antibody fragments (scFv) that are site specifically conjugated to DNA oligonucleotide barcodes, in a 1:1 manner, using a Sortase A-mediated coupling strategy. The barcoded scFvs are mixed with biotinylated serum proteins coupled to streptavidin-coated magnetic beads, and bound antibodies are detected, using NGS allowing for both a multiplex and sensitive read-out.

## Results

### The ProMIS concept

**Fig. 1:**
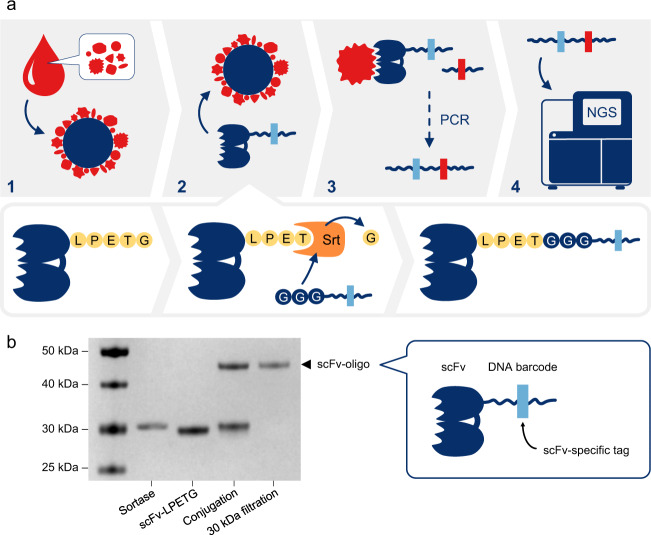
The concept of the ProMIS assay. **a** Assay principles (1–4) and the conjugation of scFv with oligonucleotides, using Sortase A (Srt). (1) Biotinylated serum proteins are captured and displayed on streptavidin-coated magnetic beads. (2) Recombinant antibody fragments (scFvs) are site-specifically conjugated 1:1 with a unique DNA oligo containing a scFv-specific tag, using a Sortase A-mediated coupling strategy. The scFv-oligos are then mixed with the beads coated with serum proteins. (3) After washing, adapter PCR is performed to equip bound scFv-oligos with a sample-specific DNA tag. (4) PCR products obtained from the combined scFv and sample tags are finally analyzed, using NGS. **b** SDS-PAGE analysis of conjugation of scFvs (containing the Sortase A recognition motif LPETG) to tri-glycine modified oligonucleotides, followed by filtration (30 kDa cutoff) to isolate only the conjugated scFv-oligos.

The concept is based on immobilization of serum proteins onto magnetic beads, followed by target binding of DNA barcoded scFv antibodies and a subsequent PCR step prior to detection by NGS (Fig. 1a). Proof-of-concept was consecutively established by selecting 17 scFv antibodies (Table 1), of which 14 have been previously reported to discriminate between serum samples derived from patients with pancreatic ductal adenocarcinoma (PDAC) and healthy controls^7^, while the three additional scFvs provide orthogonal information (unpublished data). All scFv antibodies were redesigned and produced with a C-terminal Sortase A recognition motif (LPETG), resulting in a typical yield of 1–5 mg/L. A protocol was established to conjugate the LPETG-tagged scFv antibodies to tri-glycine-modified barcode oligonucleotides, containing a tag sequence unique to each scFv. A subsequent purification step, using filtration with 30 kDa cutoff, allowed us to include only pure scFv-oligo in the assay (Fig. 1b). The use of Sortase A to conjugate oligonucleotides to scFv antibodies enables the crucial and necessary site-specific barcoding, with a 1:1 ratio. Successful conjugation and purification were confirmed by gel electrophoresis (Supplementary Fig. 1).

**Table 1:** scFv antibody fragments and corresponding target antigens.

#	Antibody	Target antigen	Target antigen (full name)
1	scFv(1)-Srt	IL-4	Interleukin-4
2	scFv(2)-Srt	MCP-1	C-C motif chemokine 2
3	scFv(3)-Srt	Lewis x	Lewis x
4	scFv(4)-Srt	Sialyl Lewis x	Sialyl Lewis x
5	scFv(5)-Srt	C1q	Complement C1q
6	scFv(6)-Srt	C5	Complement C5
7	scFv(7)-Srt	C1 est inh	Plasma protease C1 inhibitor
8	scFv(8)-Srt	Properdin	Properdin
9	scFv(9)-Srt	VEGF	Vascular endothelial growth factor
10	scFv(10)-Srt	IL-4	Interleukin-4
11	scFv(11)-Srt	C1 est inh	Plasma protease C1 inhibitor
12	scFv(12)-Srt	C5	Complement C5
13	scFv(13)-Srt	CDK2	Cyclin-dependent kinase 2
14	scFv(14)-Srt	HADH2	HADH2 protein
15	scFv(15)-Srt	APLF	Aprataxin and PNK-like factor
16	scFv(16)-Srt	MARK1-1	Serine/threonine-protein kinase MARK1
17	scFv(17)-Srt	PRKCZ	Protein kinase C zeta type

Serum samples from PDAC patients and controls were biotinylated and allowed to bind to streptavidin-coated magnetic beads. Unbound serum proteins were removed by thorough washing of the beads before a cocktail with excess of each of the 17 barcoded scFv antibodies was added to each sample for multiplex target detection. After incubation and washing, the scFv-oligos bound to the beads were PCR amplified with primers adding Illumina-compatible adapters and sample-specific indexes. The final PCR product thus contained both the scFv-specific barcode for quantification of binding events and an index to identify the sample. This allowed us to pool all the PCR products from all samples in a single NGS run on an Illumina NextSeq 500 system (Fig. 1a). The sequencing data were quality filtered and demultiplexed into counts for each scFv antibody in each sample, providing a direct digital readout. Data analysis was performed on median normalized and log2 transformed counts for supervised classification, using support vector machine (SVM) and leave-one-out (LOO) cross-validation to generate receiver operating characteristic (ROC) curves and area under the curve (AUC) values. In addition, the data were analyzed with unsupervised principal component analysis (PCA).

### Analyzing pancreatic cancer samples

**Fig. 2:**
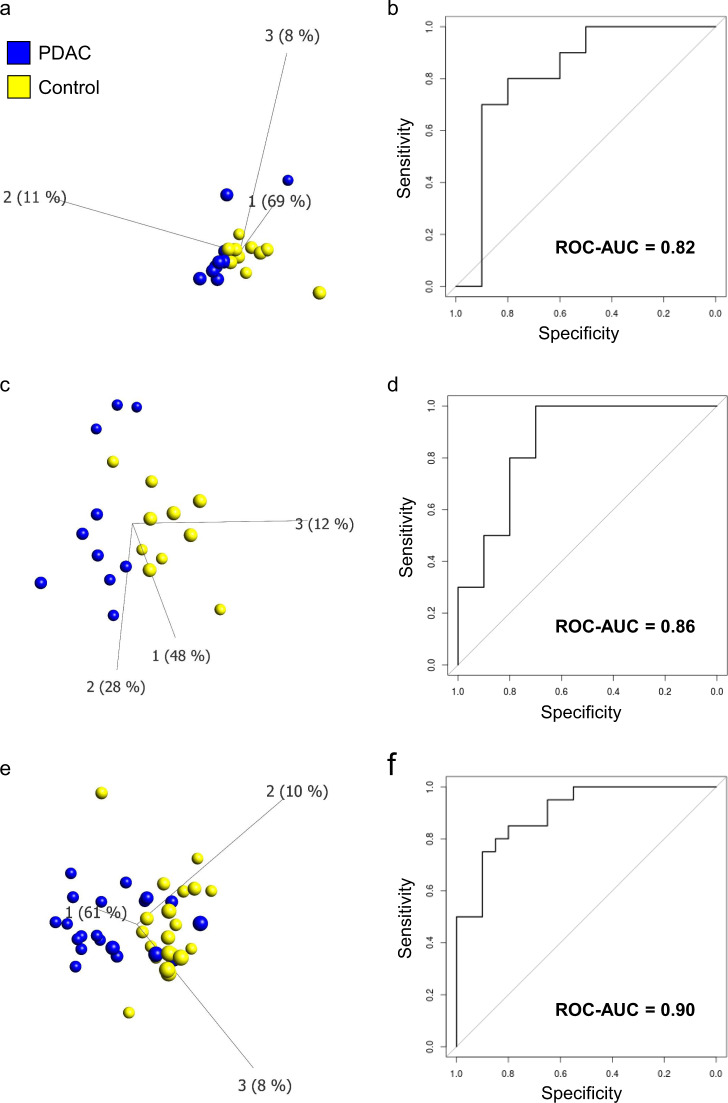
ProMIS test results from the analysis of serum samples from patients with pancreatic ductal adenocarcinoma (PDAC) compared to healthy controls. Three independent tests were performed on in total 80 distinct serum samples. Test 1: 10 PDAC samples (stage IV) versus 10 healthy controls with 16 scFv-oligos. Test 2: 10 PDAC samples (stage IV) versus 10 healthy controls with 17 scFv-oligos. Test 3: 20 PDAC samples (10 stage III and 10 stage IV) versus 20 healthy controls with 17 scFv-oligos. PCA: unsupervised visualization using principal component analysis (PCA) plotted with individual serum samples. SVM: supervised two-group classification, using support vector machine (SVM) leave-one-out cross-validation. The results are presented with receiver operating characteristic curves (ROC) and their corresponding area under the curve (AUC) values. **a** Test 1 (PCA), **b** Test 1 (SVM), **c** Test 2 (PCA), **d** Test 2 (SVM), **e** Test 3 (PCA), and **f** Test 3 (SVM).

The performance of ProMIS was demonstrated, using samples from cohorts that had previously been discriminated with ROC-AUCs of >0.90 when classifying PDAC stage I–IV versus healthy controls, using IMMray® antibody microarrays^7^. A first test was performed using 20 samples (10 PDAC stage IV and 10 healthy controls) and 16 scFv-oligos. All sequences corresponding to each specific scFv-oligo could be detected in each sample, demonstrating the functionality. The samples grouped according to disease status (healthy versus PDAC) in the unsupervised PCA plot (Fig. 2a) and resulted in a ROC–AUC of 0.82 using SVM analysis (Fig. 2b). The experiment was repeated in a second test with 20 independent samples (10 PDAC stage IV and 10 controls) analyzed with 17 scFv-oligos (one more was available at that time). Again, all specific scFv-oligos were detected, and cases and controls could be separated by PCA (Fig. 2C) and resulted in a ROC–AUC of 0.86 (Fig. 2d). The increased ROC–AUC value might be attributed to the supplementary information by the additional scFv. A final proof-of-concept was performed with 40 independent samples, including 20 PDAC, this time including samples from 10 stage III and 10 stage IV PDAC patients and 20 healthy controls. Again, all 17 specific scFv-oligos could be detected and the cases and controls were separated in PCA (Fig. 2e) and resulted in a ROC–AUC value of 0.90 (Fig. 2f).

To study the intra-assay precision of each scFv antibody, serum samples were reanalyzed using all 17 scFv-oligos with 10 replicates of each sample (Supplementary Fig. 2a). The results showed a high reproducibility with a coefficient of variation (CV) below 1% for the majority of the antibodies (Supplementary Fig. 2b).

## Discussion

This proof-of-concept study exemplifies the ability of the ProMIS platform for multiplex analysis of the human serum proteome, which provides an unparalleled approach in precision diagnostics of complex diseases. Key to the concept is the oligonucleotide barcoding of the scFv antibodies that enables the sequencing readout. The enzymatic reaction with Sortase A was found to be an effective and convenient strategy for conjugating scFv with a barcode in a 1:1 ratio under protein-compatible reaction conditions. Site-specific conjugation avoids the risk of blocking the antigen binding site, which becomes a challenge in non-specific conjugation methods. Multi-barcoding would limit the performance, which is also avoided with the 1:1 scFv:oligo conjugation principle of ProMIS.

In terms of multiplexity, the number of recombinant scFv antibodies carrying the Sortase recognition motif can easily be expanded to target virtually any antigen, using phage-display library-derived antibody fragments. The scFv antibodies used here were selected based on their combined power to discriminate PDAC versus healthy samples in antibody microarrays^7^ and demonstrated a similar performance also in ProMIS. In all three assay runs tested, each with independent serum samples, this set of scFv antibodies detected the biological differences separating the two groups with similar accuracy. Together with the low technical variation (CV < 1%), this is an indication of the robustness of the ProMIS assay.

The solution-based assay allows for easy conversion to a multi-well plate format that facilitates automation of the assay steps, which would provide consistent performance and a high sample throughput. In addition, with the PCR amplification step in combination with the NGS detection, it should be possible to tune the assay for excellent sensitivity. However, the sensitivity of the ProMIS assay will be dependent on the context of, e.g., specific biomarker signature and the number of samples that is analyzed in parallel, using a given NGS kit size (i.e. number of reads). In the present context, the sensitivity of ProMIS was shown to be comparable to an antibody microarray, using the same scFvs, demonstrating a LOD in pM–fM range^16,17^. The rapid development of NGS technology is already resulting in kits with increased coverage and decreased costs, which will catalyze how ProMIS can perform multiplexed analysis without a reduction in dynamic range.

In conclusion, we present a proof-of-concept study of the ProMIS platform, which has the potential to analyze differentially expressed proteins in serum samples with a higher throughput, multiplexity, and sensitivity, thus circumventing some of the inherent limitations with planar microarrays in precision diagnostics of complex diseases.

## Methods

### Sample preparation and capture to beads

Human serum samples from patients with PDAC (stage III or IV) and negative controls were collected by Oregon Health & Science University (OHSU), USA. All procedures were in accordance with the Institutional Review Board of Oregon Health and Science University approval. The serum proteins were biotinylated, according to a previously described protocol^18,19^. Biotinylation is widely used for protein applications, such as immobilization and functionalization^16,18,20,21^ and to verify that biotinylation of the serum sample did not considerably result in misrepresentation of the actual protein distribution we performed an analysis, using immunoprecipitation and mass spectrometry. The results showed a similar number of matching peptides for the target antigen both before and after biotinylation. In brief, 5 µL of serum samples were diluted 1:45 in phosphate-buffered saline (PBS) and labeled with 0.6 mM EZ-Link Sulfo-NHS-LC-Biotin (Thermo Fisher Scientific). Unbound biotin was removed by dialysis against PBS for 72 h using a 3.5 kDa MWCO Slide-A-Lyzer MINI dialysis unit (Thermo Fisher Scientific), changing buffer every 24 h. The labeled serum samples were aliquoted and stored at −80 °C. In the assay, 75 µL of streptavidin-coated magnetic beads, Dynabeads M-280 (Life Technologies), were used to immobilize and display 1 µL of biotinylated serum proteins.

### Generation and production of scFv-LPETG antibody fragments

Seventeen single-chain fragment variable antibodies were selected from in-house designed large phage-display libraries^22,23^. The specificity of antibodies from the libraries were previously validated with well-characterized serum samples (including spiking, blocking, and depletion of antigens) on antibody microarrays and several orthogonal methods such as mass spectrometry, enzyme-linked immunosorbent assay, and MesoScaleDiscovery cytokine assay, using various samples^16,18,24–30^.

The scFvs were used as templates in PCR reactions with primers introducing an N-terminal *Nco*I restriction endonuclease site, and a C-terminal (GS)_3_-Srt-XhoI (Srt = LPETG, Sortase tag) sequence. The generated PCR products were further used for insertion into a pET-26b(+) vector (Novagen), harboring an N-terminal pelB signal sequence and a C-terminal His_6_ tag, generating the 17 scFv gene constructs pelB-scFv-(GS)_3_-Srt-His_6_. The final gene constructs were verified by DNA sequencing.

All constructs were transformed into *Escherichia coli* BL21(DE3) cells (Merck Biosciences) and produced as previously described^18^. In brief, O/N cultures of *E. coli* were grown in 2xYT medium with appropriate antibiotics at 37 °C and induced with 1 mM isopropyl β-d-1-thiogalactopyranoside when OD reached 0.6–0.9. After O/N expression, the antibody fragments were harvested by centrifugation, lysed, and then purified using His MultiTrap 96-well filter plates (GE Healthcare).

Amicon Ultra 10K 0.5 mL centrifugal filters (Merck Millipore) were used both to change the buffer to 450 µL of Sortase ligation buffer (50 mM Tris, 150 mM NaCl, 10 mM CaCl_2_, pH 7.5)^31^ and to concentrate the purified scFvs. Purity and concentration were evaluated using 10% SDS-PAGE (Invitrogen) and a Nanodrop-1000 spectrophotometer at 280 nm (Thermo Scientific).

### Design of oligonucleotide sequences

**Fig. 3:**
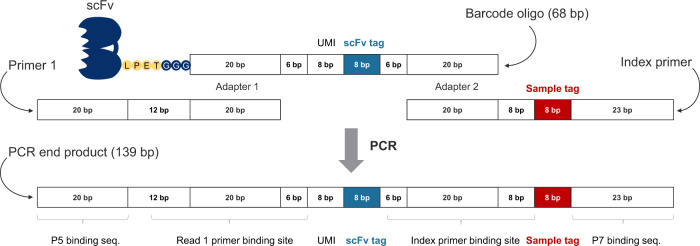
Barcode oligo design and adapter PCR. The oligonucleotide barcode contains a scFv-specific tag and is conjugated to the scFv using Sortase A. After binding to the target, the barcode is extended in both ends in the adapter PCR step with two primers. Primer 1 contains the P5 sequence needed for binding to the NGS flow cell and the Read 1 sequencing primer-binding site. The Index primer contains the index sequencing primer site, the index (sample tag), and the P7 sequence needed for binding to the NGS flow cell. The sample tag allows pooling of multiple samples and post-NGS demultiplexing of reads.

The oligonucleotide sequences (68 bp) were designed to include an 8 bp scFv-specific barcode sequence (position 35–42) used to count binding events between scFv-oligo and the target protein (Fig. 3). The sequences of all oligonucleotide barcodes are presented in Supplementary Table 1. The oligonucleotides were designed to carry a tri-glycine (G–G–G) modification in the 5′-end for the Sortase A-mediated conjugation and were purchased from Biomers AG (Ulm, Germany).

### Sortase-mediated conjugation of scFv-Srt-His6 antibodies and oligonucleotides

The oligonucleotides, carrying a tri-glycine (G–G–G) modification in the 5′-end, were used for site-specific, enzyme-dependent conjugation to scFv-Srt-His_6_. 0.2 nmol (2 µM) of scFv-Srt-His_6_ antibodies were mixed with 2 nmol (20 µM) oligonucleotides and 0.1 nmol (1 µM) high-activity mutant Sortase A in sortase ligation buffer (100 µL total reaction volume). The conjugation mixtures were incubated for 2 h at 4 °C. To purify the conjugated scFv-oligos, the conjugation mixtures were added to Amicon Ultra 30 K 0.5 mL centrifugal filters (Merck Millipore) and washed five times with 400 µL PBS. Purity and concentration was evaluated using 10% SDS-PAGE (Invitrogen) and a Nanodrop-1000 spectrophotometer at 280 nm (Thermo Scientific). A cocktail was then created by mixing 85 µL from each of the 17 purified scFv-oligos.

### ProMIS assay using barcoded scFvs

Proof-of-concept for the ProMIS assay was demonstrated, using 17 Sortase A-conjugated scFv-oligos in three experiments, two with 20 serum samples and one with 40 serum samples.

In a first step of the assay, 5 µL of biotinylated serum sample was diluted in 20 µL PBS. Five microliters of the diluted serum sample was then mixed with 75 µL of streptavidin-coated magnetic beads in 1.5 mL tubes (1 tube/sample) and incubated for 30 min in room temperature using gently agitation, according to the manufacturer’s recommendation. To remove any unbound proteins, the bead/samples were washed four times with 100 µL of washing buffer (PBS + 0.05% (v/v) Tween-20) by placing the tubes in a magnetic holder for 2 min per washing round.

Next, 32 µL of the cocktail containing a mix of all scFv-oligos was added to each tube with bead/sample and the scFv-oligos were allowed to bind their targets during an incubation at 4 °C for 2 h with gentle agitation. After three rounds of washing with washing buffer, the beads were resuspended in 50 µL of nuclease-free water and used for PCR and NGS.

### Library preparation and NGS

For adapter PCR, 8 µL of each sample was mixed with 1× Phusion Master Mix (Thermo Scientific #F-531), 0.5 µM Illumina adapter, index primer (corresponding to each sample), and nuclease-free water in a total volume of 20 µL. PCR program: 98 °C 2 min; 18 repeats of: 98 °C 20 s, 65 °C 30 s, 72 °C 30 s; 72 °C 5 min; 10 °C. PCR product purification was performed using Agencourt® AMPure XP beads according to the manufacturer’s recommendation (1.8 ratio). Positive controls contained pure barcode oligos (no scFv) and negative control (water). Quality control of purified PCR products was done using Bioanalyzer and Agilent High Sensitivity DNA kit. Five microliters of each sample was pooled, diluted, and prepared for sequencing on a NextSeq 500/550 High Output v2.5 kit (Illumina) on a NextSeq 500 sequencer (Illumina).

### NGS data analysis

NGS raw data (BCL files) generated from the NextSeq 500 were demultiplexed by the sample index reads using bcl2fastq2 Conversion Software v2.20 (Illumina) and ran through an in-house pipeline written in Java programming language to count the total number of scFv-specific tags for each sample. For our analysis we used only reads that passed the sequencer chastity filter and had base call quality for each base over Q30 (Phred Quality score)^32,33^.

Next, the counts were median normalized and log2 transformed before two-group classification using SVM LOO cross-validation to generate ROC curves and AUC values. The SVM analysis is a supervised machine learning algorithm and was performed with the R package “e1071” and a linear kernel. No prior data filtration was done before the SVM, i.e., all scFv antibodies used in the assay were also included in the analysis. The SVM finds an optimal hyperplane that separates the two groups and the classification performance is measured by the ROC–AUC value, where the value 1 would mean a perfect classifier and 0.5 a random classifier.

Data were also analyzed using PCA in Qlucore Omics Explorer 3.5 (Qlucore AB, Lund, Sweden). PCA was used as an unsupervised method to reduce the dimensionality and allow visual interpretation of the data in a 3D-plot.

### Statistics and reproducibility

When analyzing the serum samples from PDAC patients and healthy controls, no replicates were used in order to maximize the number of parallel samples. Instead, a dedicated intra-assay precision study was performed using four individual biological serum samples (two PDAC and two healthy controls) that each was divided into 10 technical replicates, where each technical replicate was analyzed with an equally sized part from a single cocktail of all 17 scFvs. Each replicate was handled in parallel in separate tubes throughout the assay and not mixed until the final pooling for analysis with a single NGS kit. The variability for each scFv is presented as Box plots in Supplementary Fig. 2a, where median value, quartiles, and range for the 10 replicates are shown for each sample. In Supplementary Fig. 2b, the same data are presented as CV values, calculated as the standard deviation divided by the mean of the 10 replicates.

### Reporting summary

Further information on research design is available in the Nature Research Reporting Summary linked to this article.

## Supplementary information

Supplementary Information

Description of Additional Supplementary Files

Supplementary Data 1

Supplementary Data 2

Reporting Summary

## Data Availability

NGS data (FASTQ files) that support the findings of this study have been deposited in Figshare at 10.6084/m9.figshare.12370106. Source data (demultiplexed reads extracted from the NGS data with the Java script) are available in Supplementary Data 1 and the processed data (median normalized and log2 transformed) are available in Supplementary Data 2.

## Code availability

The in-house pipeline written in Java for NGS data analysis is available at GitHub: https://github.com/sunnyveerla/ProMIS/blob/master/ProMIS.java.
