# Supplementary material for: Multiplex profiling of serum proteins in solution using barcoded antibody fragments and next generation sequencing
Source: Commun Biol. 2020 Jul 3;3:339. doi: 10.1038/s42003-020-1068-0 (PMC7334203; doi:10.1038/s42003-020-1068-0)
Supplement: Supplementary file 1 — Supplementary Information [file 42003_2020_1068_MOESM1_ESM.pdf]

## Supplementary Information

# Multiplex profiling of serum proteins in solution using barcoded antibody fragments and next generation sequencing

Mattias Brofelth, Anna Isinger Ekstrand, Shashank Gour, Ronnie Jansson,
My Hedhammar, Björn Elleby, Anders Kvist, Christer Wingren, Ulrika Axelsson,
Carl A.K. Borrebaeck*

Mattias Brofelth, Anna Isinger Ekstrand, Shashank Gour, Ronnie Jansson, My Hedhammar, Björn Elleby, Anders Kvist, Christer Wingren, Ulrika Axelsson, Carl A.K. Borrebaeck*

# Supplementary Figures

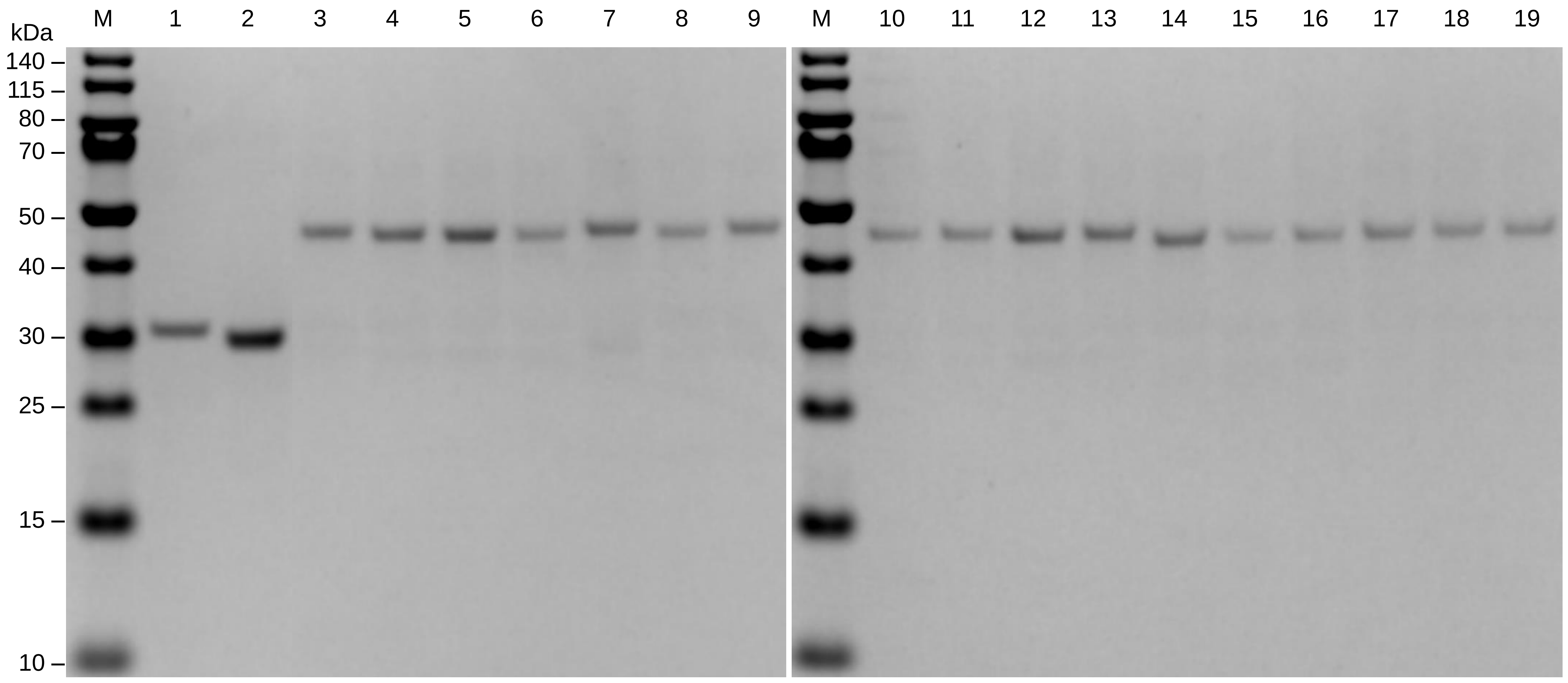


**Supplementary Figure 1.**

**SDS-PAGE of conjugated scFv antibody fragments and oligonucleotides.**

Lane 1: Sortase A enzyme.

Lane 2: Non-conjugated scFv-LPETG (scFv(1))

Lane 3-19: scFv(1)-scFv(17) conjugated to oligo barcodes, after purification with 30 kDa filter.

M = Molecular Weight Marker

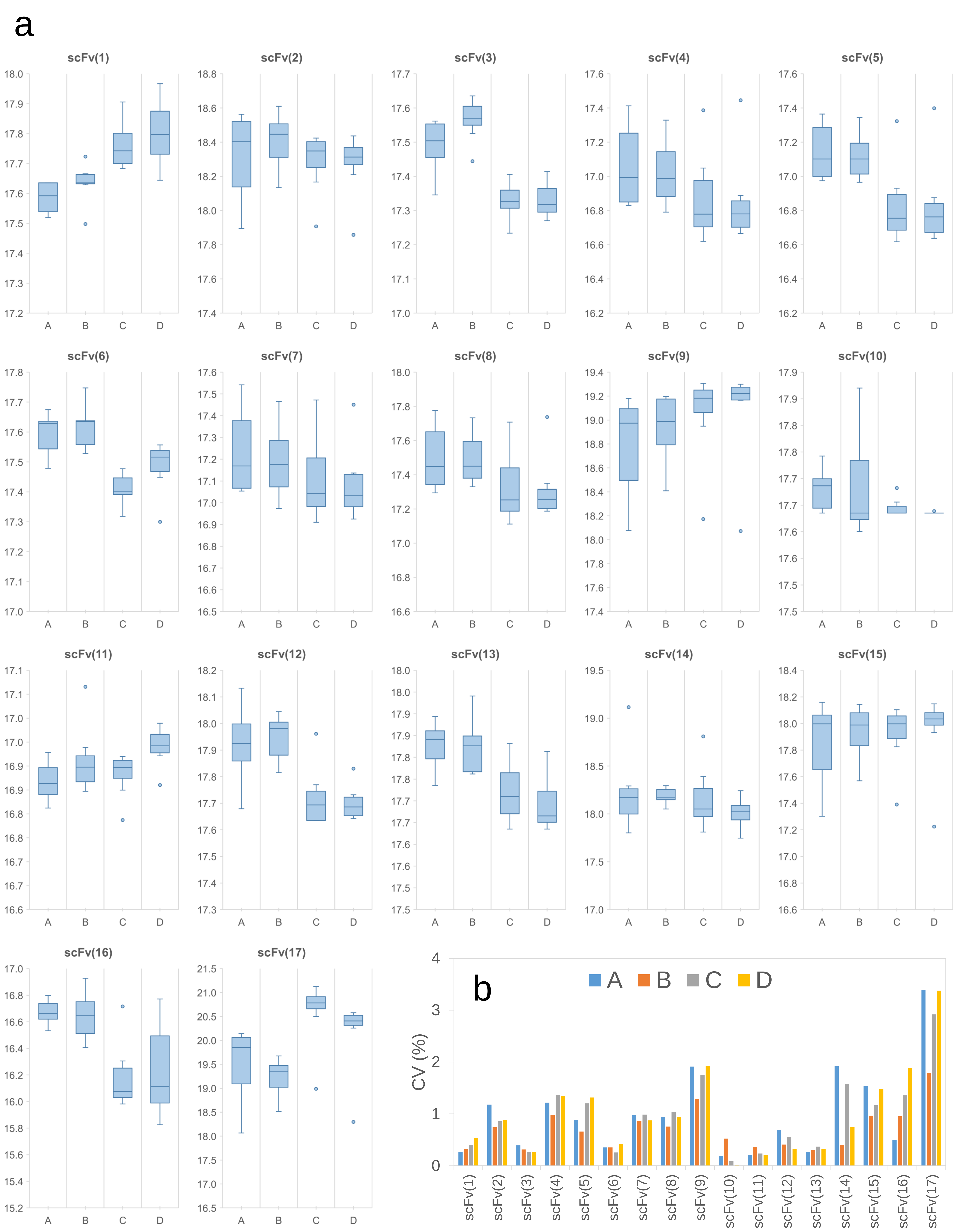


**Supplementary Figure 2. Intra-assay precision study.** Variability for each scFvs antibody was tested using n=4 biologically independent serum samples (A: Normal, B: Normal, C: PDAC stage III, D: PDAC stage IV) with 10 technical replicates each. Each of the in total 40 parallel samples were incubated with an equally sized part of a single scFv cocktail containing all 17 scFv-oligos **a)** Box plots with median, quartiles and range. Outliers are indicated by filled circles. **b)** Coefficient of variation (CV).

# Supplementary Tables

**Supplementary Table 1. Barcode oligonucleotide sequences.** 55555555 represents a sequence of 8 random bases intended to be used as an UMI (Unique Molecular Identifier), however, the UMI was not used at this stage in the development.

| | **Barcode oligonucleotide sequences** |
|---|---|
| 1 | TTCCCTACACGACGCTCTTCCGATCT55555555TGGCCTATAGATCGGAAGAGCACACGTCTGAACT |
| 2 | TTCCCTACACGACGCTCTTCCGATCT55555555ACAGTATCAGATCGGAAGAGCACACGTCTGAACT |
| 3 | TTCCCTACACGACGCTCTTCCGATCT55555555GTTAGGCAAGATCGGAAGAGCACACGTCTGAACT |
| 4 | TTCCCTACACGACGCTCTTCCGATCT55555555CACTACGGAGATCGGAAGAGCACACGTCTGAACT |
| 5 | TTCCCTACACGACGCTCTTCCGATCT55555555TGGCTAGAAGATCGGAAGAGCACACGTCTGAACT |
| 6 | TTCCCTACACGACGCTCTTCCGATCT55555555GTAGGTACAGATCGGAAGAGCACACGTCTGAACT |
| 7 | TTCCCTACACGACGCTCTTCCGATCT55555555GTTAACTGAGATCGGAAGAGCACACGTCTGAACT |
| 8 | TTCCCTACACGACGCTCTTCCGATCT55555555CACTGTACAGATCGGAAGAGCACACGTCTGAACT |
| 9 | TTCCCTACACGACGCTCTTCCGATCT55555555TGCTTACGAGATCGGAAGAGCACACGTCTGAACT |
| 10 | TTCCCTACACGACGCTCTTCCGATCT55555555ACTCAGTAAGATCGGAAGAGCACACGTCTGAACT |
| 11 | TTCCCTACACGACGCTCTTCCGATCT55555555CAGACCGTAGATCGGAAGAGCACACGTCTGAACT |
| 12 | TTCCCTACACGACGCTCTTCCGATCT55555555TGCGGAGAAGATCGGAAGAGCACACGTCTGAACT |
| 13 | TTCCCTACACGACGCTCTTCCGATCT55555555ACTACTCGAGATCGGAAGAGCACACGTCTGAACT |
| 14 | TTCCCTACACGACGCTCTTCCGATCT55555555GTATAGTCAGATCGGAAGAGCACACGTCTGAACT |
| 15 | TTCCCTACACGACGCTCTTCCGATCT55555555CAGCTCATAGATCGGAAGAGCACACGTCTGAACT |
| 16 | TTCCCTACACGACGCTCTTCCGATCT55555555ACTTAGGTAGATCGGAAGAGCACACGTCTGAACT |
| 17 | TTCCCTACACGACGCTCTTCCGATCT55555555GTAACTCCAGATCGGAAGAGCACACGTCTGAACT |
