# Supplementary material for: Multiplex profiling of serum proteins in solution using barcoded antibody fragments and next generation sequencing
Source: Commun Biol. 2020 Jul 3;3:339. doi: 10.1038/s42003-020-1068-0 (PMC7334203; doi:10.1038/s42003-020-1068-0)
Supplement: Supplementary file 2 — Description of Additional Supplementary Files [file 42003_2020_1068_MOESM2_ESM.pdf]

Description of Additional Supplementary Files

File Name: **Supplementary Data 1**
Description: Source NGS data

File Name: **Supplementary Data 2**
Description: Processed NGS data
