# Supplementary material for: Multiplex profiling of serum proteins in solution using barcoded antibody fragments and next generation sequencing
Source: Commun Biol. 2020 Jul 3;3:339. doi: 10.1038/s42003-020-1068-0 (PMC7334203; doi:10.1038/s42003-020-1068-0)
Supplement: Supplementary file 5 — Reporting Summary [file 42003_2020_1068_MOESM5_ESM.pdf]

nature research

Corresponding author(s): Carl Borrebaeck

Last updated by author(s): May 29, 2020

Reporting Summary

Nature Research wishes to improve the reproducibility of the work that we publish. This form provides structure for consistency and transparency in reporting. For further information on Nature Research policies, see Authors & Referees and the Editorial Policy Checklist.

Statistics

For all statistical analyses, confirm that the following items are present in the figure legend, table legend, main text, or Methods section.

n/a	Confirmed	
☐	☒	The exact sample size (n) for each experimental group/condition, given as a discrete number and unit of measurement
☐	☒	A statement on whether measurements were taken from distinct samples or whether the same sample was measured repeatedly
☒	☐	The statistical test(s) used AND whether they are one- or two-sided<br>*Only common tests should be described solely by name; describe more complex techniques in the Methods section.*
☒	☐	A description of all covariates tested
☒	☐	A description of any assumptions or corrections, such as tests of normality and adjustment for multiple comparisons
☐	☒	A full description of the statistical parameters including central tendency (e.g. means) or other basic estimates (e.g. regression coefficient) AND variation (e.g. standard deviation) or associated estimates of uncertainty (e.g. confidence intervals)
☒	☐	For null hypothesis testing, the test statistic (e.g. F, t, r) with confidence intervals, effect sizes, degrees of freedom and P value noted<br>*Give P values as exact values whenever suitable.*
☒	☐	For Bayesian analysis, information on the choice of priors and Markov chain Monte Carlo settings
☒	☐	For hierarchical and complex designs, identification of the appropriate level for tests and full reporting of outcomes
☒	☐	Estimates of effect sizes (e.g. Cohen's d, Pearson's r), indicating how they were calculated

Our web collection on statistics for biologists contains articles on many of the points above.

Software and code

Policy information about availability of computer code

Data collection	NextSeq 500 software (Illumina)
Data analysis	Sequencing Analysis Viewer (SAV) 1.9.1 (Illumina), bcl2fastq2 Conversion Software v2.20 (Illumina), R package "e1071" (for SVM classification), Microsoft Excel, Qlucore Omics Explorer 3.5.<br>In-house pipeline written in Java for NGS data analysis is available at GitHub: https://github.com/sunnyveerla/ProMIS/blob/master/ProMIS.java

For manuscripts utilizing custom algorithms or software that are central to the research but not yet described in published literature, software must be made available to editors/reviewers. We strongly encourage code deposition in a community repository (e.g. GitHub). See the Nature Research guidelines for submitting code & software for further information.

Data

Policy information about availability of data

All manuscripts must include a data availability statement. This statement should provide the following information, where applicable:

- Accession codes, unique identifiers, or web links for publicly available datasets
- A list of figures that have associated raw data
- A description of any restrictions on data availability

NGS data (FASTQ files) that support the findings of this study have been deposited in Figshare at http://dx.doi.org/10.6084/m9.figshare.12370106. Source data (demultiplexed reads extracted from the NGS data with the Java script) is available in Supplementary Data 1 and the processed data (median normalized and log 2 transformed) is available in Supplementary Data 2.

Field-specific reporting

Please select the one below that is the best fit for your research. If you are not sure, read the appropriate sections before making your selection.

☒ Life sciences ☐ Behavioural & social sciences ☐ Ecological, evolutionary & environmental sciences

For a reference copy of the document with all sections, see nature.com/documents/nr-reporting-summary-flat.pdf

Life sciences study design

All studies must disclose on these points even when the disclosure is negative.

Sample size	This is a concept study with no interventional aspects not requiring any power calculation<br>n(total)=80 independent serum samples. n(test1)=10+10, n(test2)=10+10, n(test3)=20+20<br>Cases (PDAC patients) were compared to an equal number of healthy controls.
Data exclusions	No data was excluded.
Replication	To maximize the number of independent samples when comparing the groups, no replicates were used in the first 3 experiments.<br>10 technical replicates per reanalyzed sample were instead used in the separate technical intra-assay experiment.
Randomization	Serum samples were randomized by disease status.
Blinding	Known disease status was needed for SVM classification.

Reporting for specific materials, systems and methods

We require information from authors about some types of materials, experimental systems and methods used in many studies. Here, indicate whether each material, system or method listed is relevant to your study. If you are not sure if a list item applies to your research, read the appropriate section before selecting a response.

Materials & experimental systems

n/a	Involved in the study	
☐	☒	Antibodies
☒	☐	Eukaryotic cell lines
☒	☐	Palaeontology
☒	☐	Animals and other organisms
☒	☐	Human research participants
☒	☐	Clinical data

Methods

n/a	Involved in the study	
☒	☐	ChIP-seq
☒	☐	Flow cytometry
☒	☐	MRI-based neuroimaging

Antibodies

Antibodies used	scFv antibody fragments selected from in-house phage-display library
Validation	In-house validation: scFvs from the library has passed stringent phage-display selection and several clones have also been validated using samples with known target concentrations, spiked or depleted samples, as well as orthogonal methods such as MS, ELISA, Meso Scale Discovery (MSD) and cytometric bead assay.
